# Supplementary material for: Conceptual frameworks for understanding the acceptability and feasibility of the minimally invasive autopsy to determine cause of death: Findings from the CADMIA Study in western Kenya
Source: PLoS One. 2020 Dec 14;15(12):e0242574. doi: 10.1371/journal.pone.0242574 (PMC7735626; doi:10.1371/journal.pone.0242574)
Supplement: S1 Appendix — (DOCX) [file pone.0242574.s001.docx]

**S1 Appendix: Nodes used in Analysis**

1. **DEATH**
   1. **Rituals and norms. Rituals:** Series of actions performed around the corpse and relatives (e.g., widow) according to a prescribed order. **Norm:** Standard or pattern that is typical or expected.

Includes work, sexual relations, purification, food, taboos.

- 1. **Procedures:** Series of actions around death. Includes handling of the corpse, communication of the death, informed consent.
  2. **Behaviours, reactions and attitudes:** The way in which one acts or conducts oneself, especially towards others. e.g., crying, showing anger, etc. Includes Health Community Workers as well as family of deceased.
  3. **Bureaucracy**: Administrative procedures around death. Includes death certificates, inheritances processes and other legal documents.
  4. **Cause of death**
     1. **Specific causes:** Particular cause/s attributed to the death. Reason why death has occurred (ex. a particular illness, an action, an effect)
        1. **HIV/AIDS** mentioned specifically
        2. **Witchcraft/*chiraa*/etc.** mentioned as CoD
     2. **Willingness to know**: Any instance in which there is a clear statement about wanting to know about the cause of death (a relatives’ one or the family to know the cause of his/her own death), regardless of the method.
     3. **Lack of willingness to know CoD:** Any instance in which there is a statement about NOT wanting the know the cause of death of a relative or another person, regardless of method

1. **MINIMALLY INVASIVE AUTOPSIES (MIA):** Use targeted small diagnostic biopsies of key organs. The biopsies are examined and analyzed in a laboratory for pathological and microbiological diagnosis. No large cuts are needed and no organs are removed.
   1. **Reasons for accepting MIA:** S/E
      1. **Compensation,** mention of compensation provided as a reason to accept MIA, e.g. financial benefit in terms of catering for some expenses like coffin, hospital/mortuary bill, etc.
   2. **Reasons for MIA refusal:** S/E. Includes incompatibility with rituals and norms.
   3. **Willingness to consent (or not) MIA**: S/E, may include “conditions” under which MIA would be acceptable
      1. **Logistics related to MIA acceptability:** Statements made regarding MIA acceptability related to timing, where, who would conduct etc.
2. **AUTOPSY:** A post-mortem examination of the body, using dissection techniques and exposure of the vital organs, to discover the cause of death or the extent of disease.
   1. **Reasons for accepting autopsy:** S/E
      1. **Compensation,** mention of compensation provided as a reason to accept MIA
   2. **Reasons for autopsy refusal:** S/E. Includes incompatibility with rituals and norms.
   3. **Willingness to consent autopsy**: S/E
      1. **Logistics related to MIA acceptability:** Statements made regarding autopsy acceptability related to timing, where, who would conduct etc.
   4. Lack of willingness to consent autopsy: S/E
   5. **Previous autopsy study:** an autopsy study which was conducted in the study area between 2012-2013.
      1. **Statements pertaining to positively influence the previous autopsy study conducted in the area**
      2. **Statements pertaining to negatively influence the previous autopsy study**
3. **VERBAL AUTOPSIES (VA):** Method of obtaining as much information as possible about a deceased person by asking questions to family and others who can describe the mode of death and circumstances preceding death. Used especially in settings and situations in which postmortem pathologic examination is not feasible.
4. **CORPSE: Self-explanatory** (S/E)
   1. **Physical Characteristics** [man, woman, child, age, albinism…]
   2. **Spirit/Soul:** The non-physical part of a person manifested as an apparition after their death. A ghost. Immaterial part of a human being.
      1. **Spirit “not at rest”:** Specific comments about circumstances that may lead a spirit to not be at rest and/or to haunt the survivors
5. **PEOPLE - SOCIAL ENTITIES:** Members of communities, individuals and collectives
   1. **Family:** Relatives. Includes wife, husband, partner, sons, daughters, father, mother, brothers, sisters, in-laws, cousins, aunts, uncles, etc.
   2. **Neighbours:** S/E
   3. **Community leaders, elders:** Prominent member of the community who knows the ritual and ethnic norms and can influence the opinion of the community.
   4. **Religious leaders:** Anyone who rules or guides a religious organization, a church, a mosque, a cult; or have influence on others within his/her congregation.
   5. **Religious peers:** Member/s of the same religious organization, church or cult.
   6. **Health workers and Health authorities:** S/E. Within the formal health system. Includes information such as "The hospital says that..."
      1. Distrust of / negligence by health care workers (may overlap with other themes)
   7. **Traditional healers:** People, recognized by the community, that use non-western forms of cure (including herbalists, spiritualists, etc). Also anyone known to use plants with a therapeutic purpose, without being labelled “traditional healer.”
   8. **Traditional Midwifes:** Person from the community, usually a woman, who assist women during childbirth and it is not part of the formal health system.
   9. **Funeral home staff.** S/E. Includes other embalmers (e.g. those who come to homes to conduct)
   10. **Service providers:** People who have business related to/around death. Includes carpenters who makes the coffins, drivers who transport the corpse, people who sells flowers, people who sells food, etc.
   11. **Police, governmental/municipal authorities/administration:** S/E, Includes comments about legal workers in the case of suicides
   12. **Decision maker:** Who has the decision-making power in relation to death procedures and events, MIAS and CDA. Include attributes of decision-makers, eg role on income, specific family roles etc.
   13. **KEMRI and CADMIA staff:** S/E
   14. **Men:** S/E
   15. **Women:** S/E
       1. Maternal deaths
   16. **Age**
       1. **Children** (neonates to age 18/age of adulthood)
       2. Adult (any one above 18 years, also depends on the spaekers definition)
       3. **Elderly** (as defined by speaker)
6. **PLACES:**S/E
   1. **Home:** Place of residence. A place where someone has settled for a long-term basis.
   2. **Neighbourhood** Area surrounding a particular place, in relation with “home”. Includes “community” and information such as (example): “At the community, people used to…”
   3. **Work place:** S/E, place of employment, etc. Excludes all places defined as “Health facility” (Node 7.7).
   4. **Cemetery:** Burial ground. Familiar or municipal.
   5. **Church/Mosque:** A building/space used for public worship.
   6. **Funeral Home and Morgue** Establishment/business that made the necessary arrangements before and during the funeral.
   7. **Health facility:** S/E. Includes health posts, out-patients consultation rooms, district and referral hospitals, mortuary services within the health facilities.
   8. **KEMRI**: Comments related to Kenya Medical Research Institute
7. **TIMING**: Period in time with reference to the death event. Can be actual period or reported period.
   1. Before death
   2. Immediately after death
   3. After death
      1. $\leq$ 24 hours after death
      2. 1-10 days after death
      3. ≥ 11 days after death
   4. **Communication of MIA results**
      1. Period waiting for results – how long should it from the time of taking the corpse for MIA to giving back results to next-of-kin
      2. Who to communicate MIA results to next-of-kin
      3. Who should be given the results among the next-of-kin
8. **COSTS AND RESOURCES:** Expected or actual expenses related to death events, in money or goods/Support and help from community, family, friends, in money or goods. Related costs of Autopsies and MIAs.
   1. **Sustainability of MIA:** Comments statements about the long-term ability to support MIA and how this should be done
