# Supplementary material for: Conceptual frameworks for understanding the acceptability and feasibility of the minimally invasive autopsy to determine cause of death: Findings from the CADMIA Study in western Kenya
Source: PLoS One. 2020 Dec 14;15(12):e0242574. doi: 10.1371/journal.pone.0242574 (PMC7735626; doi:10.1371/journal.pone.0242574)
Supplement: Attachment — Submitted filename: Response to Reviewers.docx [file pone.0242574.s003.docx]

| **Location** | **Comment** | **Response** |
| --- | --- | --- |
| Overall | Well written paper but there are areas which need clarification, refer to the in-text comments. I was particularly looking forward to whether there were some sort of comparative reports mentioned between the 3 groups of which one group was in the immediate phase of grieving of which again, there was a large sample variation which also needs explanation and how the authors will control bias on reported statements given the sample variation. This needs clarity.) | Thank you for this comment. Please see below/in-text for the in-text comments  Re comparisons: The difference in sample is described below,  We did review the transcripts for the three groups, namely: next of kin 1) within 24 hours; 2) within one week; 3) within 30-40 days after death, and did not find discernable differences in the perspective that was offered, therefore this was not a focus of the manuscript. We were able to have longer, more in-depth conversations with people who were a bit further away from the deaths, which very much informed the approach used in future research.  Moreover, the reason behind the three groups, was to maximize the possibilities of feasible data collection despite the grieving status of the participants. The team across the sites decided to start the study by interviewing next of keen within 30 to 40 days after the death and gradually moving to interviews closer to the time of death. This helped interviewers gain familiarity with interviewing grieving family members and allowed for gradual exposure to family’s distress with increasing levels of distress coupled with increasing capacity to deal with such situations. The categorization was not made for the purposes of comparison, however in another study (multicountry) it was possible to explore potential differences in acceptability across the different groups. (reference, [1]). |
|  |  |  |
| P3 | **Reviewer**: Are you sure MIA will not face some of this challenges too? Will it be point of care/death test or sent to other centres? | Thank you for the observation. Indeed, the focus of the manuscript is to address many of the challenges that MIA can face.  Use of ‘may’ in the following paragraph is an indication that MIA is not excepted, however, the degree may be less compared to CDA: “Less invasive approaches as an alternative to CDA may be more feasible as well as more acceptable in LMIC [2].”  With regard to the question posed -- Point of care testing is extremely limited in scope (pathogens covered etc), and therefore the majority of testing is conducted in laboratories. The references for MIA explain methods thoroughly. Furthermore, testing in the laboratory allows for much better confidentiality. |
| P 6/Results | Reviewer: Any explanation for this huge difference in sample sizes between this three groups? | Thank you for the question. The difference reflects both design and logistics.  A minimum sample size of 30 participants for each target group (next of kin, key informants) was settled in the study ‘protocol, taking into consideration that saturation point could be achieved before or after the minimum sample size is reached. Regarding the sub-target group of next of kin within 0-24 hours after the death, the following challenges for contacting them and collecting data were faced, but we consider that saturation point for the whole next of kin target group was reached. Please, kindly see below these challenges:  As the majority of the study staff are from the community, it was well understood that asking families for a “research-oriented” conversation at the time of death would be challenging. This reflects both the emotional challenges but also the very real logistical issues that families face in dealing with a death. We did have focused conversations with families at the time immediately after death, but these were few in numbers.  As well, the reporting system for deaths by the village reporters to study staff was not always immediate, therefore some deaths were not reported as soon as they occurred. Sometimes village reporters would learn of the death information late especially those that occurred in far-away hospitals (and in towns) thus reporting late. Our village based staff also faced challenges like phone running out of power to send text messages or make calls or challenge of airtime    We employed the HDSS Verbal Autopsy tracking system to get information about deaths that occurred. However, this was helpful for next-of-kin within more than a week and one month after death cases. Together these systems allowed us to find most deaths in the absence of a fully incentivized early reporting network (which was developed later on.) |
| Overall, reviewer 2 | 1.Please arrange all heading and description in a orderly manner. In some page the heading is there but description is in next page. | Thank you, we have corrected the pagination. We are aware that this will change when formatted for the journal as well. |
|  | 2. There are some typos in the document. Please correct those typos. | Thank you, we have reviewed the manuscript and we have re-run the spell-checker |
|  | 3. During formulating questionnaire and checklist of qualitative part did you go for back to back translation, which should be unique one | We are not entirely clear on the meaning of the question, but we did conduct back translation. The majority of the interviews were conducted in Dholuo, the local language or Swahili, transcribed in the local language, translated into English and then back-translated by another of the researcher when quotes were selected. In several cases, in-depth discussion of the appropriate English translation was conducted. |

**Reference**

1.	Maixenchs M, Anselmo R, Zielinski-Gutiérrez E, Odhiambo FO, Akello C, Ondire M, et al. Willingness to know the cause of death and hypothetical acceptability of the minimally invasive autopsy in six diverse African and Asian settings: a mixed methods socio-behavioural study. PLoS medicine. 2016;13(11):e1002172.

2.	Nyamthimba P, Gacheri S, Laserson K, Agaya J, Cain K, Sitienei J, et al. Experiences in Implementing Verbal Autopsy And Postmortem study For Measuring Tuberculosis Mortality In Kenya. in prep. 2016.
